# Phenotype and functional evaluation of *ex vivo *generated antigen-specific immune effector cells with potential for therapeutic applications

**DOI:** 10.1186/1756-8722-2-34

**Published:** 2009-08-06

**Authors:** Shuhong Han, Yuju Huang, Yin Liang, Yuchin Ho, Yichen Wang, Lung-Ji Chang

**Affiliations:** 1 Department of Molecular Genetics and Microbiology, College of Medicine, University of Florida, Gainesville, FL 32610-0266; 2 Vectorite Biomedica, Inc., Taipei, Taiwan, Republic of China

## Abstract

*Ex vivo *activation and expansion of lymphocytes for adoptive cell therapy has demonstrated great success. To improve safety and therapeutic efficacy, increased antigen specificity and reduced non-specific response of the *ex vivo *generated immune cells are necessary. Here, using a complete protein-spanning pool of pentadecapeptides of the latent membrane protein 2A (LMP2A) of Epstein-Barr virus (EBV), a weak viral antigen which is associated with EBV lymphoproliferative diseases, we investigated the phenotype and function of immune effector cells generated based on IFN-γ or CD137 activation marker selection and dendritic cell (DC) activation. These *ex vivo *prepared immune cells exhibited a donor- and antigen-dependent T cell response; the IFN-γ-selected immune cells displayed a donor-related CD4- or CD8-dominant T cell phenotype; however, the CD137-enriched cells showed an increased ratio of CD4 T cells. Importantly, the pentadecapeptide antigens accessed both class II and class I MHC antigen processing machineries and effectively activated EBV-specific CD4 and CD8 T cells. Phenotype and kinetic analyses revealed that the IFN-γ and the CD137 selections enriched more central memory T (Tcm) cells than did the DC-activation approach, and after expansion, the IFN-γ-selected effector cells showed the highest level of antigen-specificity and effector activities. While all three approaches generated immune cells with comparable antigen-specific activities, the IFN-γ selection followed by *ex vivo *expansion produced high quality and quantity of antigen-specific effector cells. Our studies presented the optimal approach for generating therapeutic immune cells with potential for emergency and routine clinical applications.

## Background

The key to the success of immune cell therapy is to increase specific and decrease non-specific immune response. *Ex vivo *expanded antigen-specific T cells targeting cytomegalovirus (CMV), Epstein-Barr virus (EBV) and adenovirus have been successfully applied to treating hematopoietic stem cell or solid organ transplant patients who have developed post-transplant viral diseases. [1,2] The labor-intensive and time-consuming process of isolating and expanding antigen-specific immune cells, however, has hindered common practice of this medical advent.

Donor leukocyte infusion has attained a response rate of >50% in treating post-transplant infections or cancer relapse of hematopoietic stem cell transplant (HSCT) patients. [3-5] Non-specific leukocyte infusion, however, may cause severe graft-versus-host disease (GvHD) with high morbidity and mortality. Adoptive transfer of antigen-specific T cell clones combined with clinical regimens to increase immune cell homeostasis has illustrated high response rate (50%) in melanoma patients. [6-8] While the amplified T cell clones display increased antigen-specific effector functions, the *ex vivo *expansion process takes longer than 4–6 weeks, which often results in terminal differentiation of the T cells with reduced *in vivo *proliferation potential. [9]

A few approaches have been developed to directly isolate antigen-specific immune cells, such as the use of MHC-peptide multimers and selection of IFN-γ-secreting cells with affinity-magnetic beads. [10-14] Recently, CD137 (4-1BB), a member of the tumor necrosis factor receptor family, has been reported to be a suitable surface marker for antigen-specific T cell isolation. [15] Although both the IFN-γ and the CD137 selection methods generate only a small number of immune effector cells, these cells may be further expanded in culture to obtain more functional cells. [12]

Detailed phenotype and functional characterizations of these *ex vivo *prepared immune effector cells are necessary to facilitate their clinical applications. Here, we refined and compared three of the state-of-the-art immune effector cell preparation approaches, the IFN-γ and the CD137 selection methods for emergency preparation of therapeutic cells, and a DC-immune cell coculture method for the expansion of antigen-specific immune cells. We targeted EBV using LMP2A pentadecapeptides as antigens because EBV-associated lymphoproliferative disorders represent one of the most severe problems in HIV/AIDS patients and transplantation patients. The IFN-γ-selected cells showed an increased ratio of CD4 or CD8 effector cell population depending on the donor, whereas the CD137 selection method enriched a higher ratio of CD4 T cells regardless of the donor's T cell dominance response. Both of the rapid protocols yielded more central memory and effector memory T cells than did the DC-activation method. Our detailed side-by-side comparison concludes that IFN-γ selection followed by *ex vivo *expansion represents the preferred method for the generation of antigen-specific immune effector cells with potential for clinical applications.

## Methods

### Peripheral blood mononuclear cells (PBMC) and B lymphoblastoid cell lines (BLCL)

Healthy donors' buffy coats were obtained from Civitan Blood Center (Gainesville, FL, USA). PBMC were prepared by gradient density centrifugation in Ficoll-Hypaque (GE Healthcare Bio-Sciences AB, NJ, USA) as previously described. [16] Viability was determined by trypan blue staining. Autologous B lymphoblastoid cell line (BLCL) was generated by transforming peripheral blood B lymphocytes with EBV as described previously. [17] The BLCL were continuously propagated in RPMI 1640 medium supplemented with 2 mM L-glutamine, 100 μg/ml streptomycin, 100 IU/ml penicillin and 10% heat inactivated fetal bovine serum (FBS) at 37°C with 5% CO_2_.

### Peptides

The mixtures of 11 amino acid overlapping pentadecapeptides (122 peptides) spanning the entire 497 amino acids (NCBI Accession number P13285) of LMP2A of the EBV (human herpesvirus 4, strain B95-8) and the Wilms' tumor antigen (WT1, 449 amino acids, 110 peptides) were purchased from JPT Peptide Technologies GmbH (Berlin, Germany).

### Preparation of 2 day and 5 day dendritic cells (DC)

PBMC were plated into 6-well plate at 1 × 10^7 ^cells/well and adhered for 2 hours in AIM-V (Gibco-BRL, CA, USA). The non-adherent cells were removed gently and frozen as source of lymphocytes for co-culture use. Adherent monocytes were cultured in AIM-V supplemented with 50 ng/ml of GM-CSF and 25 ng/ml IL-4 (eBiosource International, Inc. Camarillo, CA, USA). For the generation of 2 day DC, the adherent cells were cultured with GM-CSF and IL-4 for 24 h and incubated for another 24 h with TNFα (50 ng/ml), IL-1β (10 ng/ml), IL-6 (10 ng/ml, all from R&D systems, MN, USA) and PGE2 (1 uM, Sigma-Aldrich, MO, USA) to induce maturation. For the generation of 5 day DC, cells were cultured with GM-CSF and IL-4 for 5 days. On day 3, half of the medium was replaced with fresh medium containing GM-CSF and IL-4. On day 5, the immature DC were induced into maturation with TNFα (50 ng/ml), LPS (1 μg/ml, Sigma-Aldrich) and IFN-γ (50 ng/ml, R&D systems).

### Isolation of antigen-specific IFN-γ secreting or CD137 positive cells

PBMC were resuspended in AIM-V plus 5% human AB serum at 1 × 10^7 ^cells/ml and mixed with pentadecapeptides for EBV-LMP2A (10 ug/ml), mouse anti-human CD28 antibody (Ab, 1 ug/ml, eBioscience, San Diego, USA) and human β2-microglobulin (1 ug/ml, Sigma) to enhance antigen presentation and costimulation. The cells were incubated in a 37°C humidified incubator for 3–13 hours. IFN-γ secreting cells were enriched with the IFN-γ Catch Reagent and CD137 positive cells with PE-conjugated monoclonal anti-CD137 Ab (clone 4B4-1), followed by affinity isolation using anti-PE microbeads according to the manufacturer's instruction (Miltenyi Biotech Inc. Auburn, CA, USA).

### Generation of DC-activated antigen-specific immune cells

DC-activated antigen-specific immune cells were generated as previously described. [16] In brief, mature 2 day DC were loaded with LMP2A peptides (2.5 ug/ml) for 2 h and irradiated (20 Gy, or 2,000 rads). The antigen-pulsed DC were cocultured with autologous non-adherent PBMC at a ratio of 1:20 in AIM-V with 5% human AB serum. On day 3, half of the medium was replaced with fresh medium supplemented with IL-2 (12.5 U/ml), IL-7 (5 ng/ml) and IL-15 (20 ng/ml, all from Gentaur, Aachen, Germany). Half of the medium was replaced with fresh medium with cytokines every other day.

### Multi-color flow cytometry

Mature DC were analyzed using a four-color panel of monoclonal Ab including PE-anti-CD14, FITC-anti-HLA-DR, PE-anti-CD86, APC-anti-CD1a, PE-anti-CD 83, APC-anti-CD40 and FITC-anti-HLA-I (BD Biosciences, San Jose, CA, USA), APC-anti-DC-SIGN and PE-cy7-anti-CD11c (eBioscience), and incubated for 30 min at 4°C. Isotype-matched antibodies were used for controls. The Ab-labeled cells were washed twice with PBS containing 1% FBS and analyzed with FACSCaliber or FACSAria using FACSDiva software (BD Biosciences) and Flowjo software (Tree Star, Inc., Ashland, OR, USA). For memory T cell analysis, the cells were stained with PE-anti-IFN-γ or PE-anti-CD137, in combination with APC-anti-CD4 (clone RPA-T4), Pacific blue-anti-CD8 (clone RPA-T8), FITC-anti-CD45RA (clone HI100), PE-cy7-anti-CCR7 (clone 3D12), Percp-cy5.5-anti-CD28 (clone 293, all from BD Biosciences) and Alexa-fluo 750-anti-CD27 (clone O323, eBioscience) at 4°C for 30 min and washed twice with PBS containing 1% FBS. The percentage of different T cell subsets was analyzed using FACSAria with FACSDiva and Flowjo softwares.

### Immune effector assays: CD107a degranulation and intracellular cytokine staining

These assay were performed as described. [18] Briefly, 2 × 10^5 ^LMP2A-specific T cells were stimulated for 5 h in a 96-well plate with irradiated (20 Gy) antigen-loaded autologous DC. Monensin A (Sigma-Aldrich) and FITC-conjugated Abs for CD107a or isotype matched Abs (BD Pharmingen, San Diego, CA, USA) were added 1 hour after stimulation and incubated for 5 hours. Cells were then stained with Abs against CD4 and CD8 and fixed, permeabilized with Cytofix/Cytoperm solution and stained with Ab against IFN-γ (all from BD Pharmingen) at 4°C for 20 min. Unrelated peptide group was included as a negative control for spontaneous CD107a expression and/or cytokine production.

### Detection of peptide-specific CD8+ T cells by MHC multimer analysis

Peptide-major histocompatibility complex (MHC)-pentamer conjugate specific for EBV-LMP2A/TYGPVFMCL (HLA-A*2402 restricted) was purchased from Proimmune (Springfield, VA, USA). T cells were incubated with PE-labeled peptide MHC-pentamer at room temperature for 10 min, washed and stained with APC-anti-CD3 and FITC-anti-CD8 Ab (BD Pharmingen) on ice for 30 min, and analyzed using FACSAria. At least 1 × 10^5 ^events were collected for each sample.

### T cell proliferation assay with carboxy-fluorescein diacetate succinimidyl ester (CFSE) staining

The CFSE-based proliferation assay was performed as previously described [19]. Briefly, LMP2A-specific T cells were washed and labeled with 1 uM CFSE (Molecular Probes, Inc., Eugene, OR, USA). The labeled cells were washed and plated into 96-well U-bottom wells at 1 × 10^5 ^cells per well. Autologous DC were loaded with peptides (2.5 ug/ml) for 2 hours and irradiated (20 Gy). The irradiated DC were added to the CFSE-labeled T cells at a ratio of 1:20 and cultured in AIM-V with 5% human AB serum. After 4 days, cells were harvested and analyzed with flow cytometry.

### Antigen-specific cytotoxicity assay of immune effector cells

The immune cell cytotoxicity assay was based on Jedema et al. with minor modifications. [20] The target cells were washed with PBS, and labeled with 1 uM CFSE (Molecular Probes) at 5 × 10^6 ^cells per ml at 37°C for 15 minutes. The reaction was stopped with the addition of 10 volumes of complete RPMI containing 10% FCS, followed with a 30 min incubation at 37°C. After two washes, the CFSE-labeled target cells were resuspended in AIM-V containing 5% human AB serum. The cell concentration was adjusted to 1 × 10^5 ^cells/ml before plating into 96-well microtiter plates at 100 ul/per well. The effector cells were then mixed with target cells at a ratio of 1:1. The plates were incubated in a humidified atmosphere of 5% CO_2 _and 37°C. The target cells included irradiated (20 Gy) autologous DC loaded with LMP2A peptides or control WT1 peptides, and irradiated (100 Gy) autologous BLCL. The effector cells included LMP2A peptide-stimulated IFN-γ-selected cells, LMP2A pulsed DC-expanded effector cells, LMP2A peptide-stimulated IFN-γ-negative PBMC, and control PBMC. After 6 hour of incubation, the cells were mixed with 10,000 Flow-Count Fluorospheres (BD Pharmingen) and followed by flow cytometry analysis. To stain for dead cells, 7-AAD (10 ug/ml) was added and incubated for 30 min on ice. For each sample, 5,000 microbeads were acquired, facilitating the calculation of absolute numbers of target cells. The percentage of survival was determined as the following:



(R refers to different CTL ratio groups; R = 0 refers to the CTL = 0 control)

### Statistical analysis

Data were analyzed using GraphPad Prism 4 analysis software (GraphPad Software Inc. San Diego, CA) and Student's *t-test*. A 2-sided P value of less than 0.05 was considered statistically significant.

## Results

### Both IFN-γ and CD137 are effective markers for the isolation of antigen-specific immune effector cells

**Figure 1:**
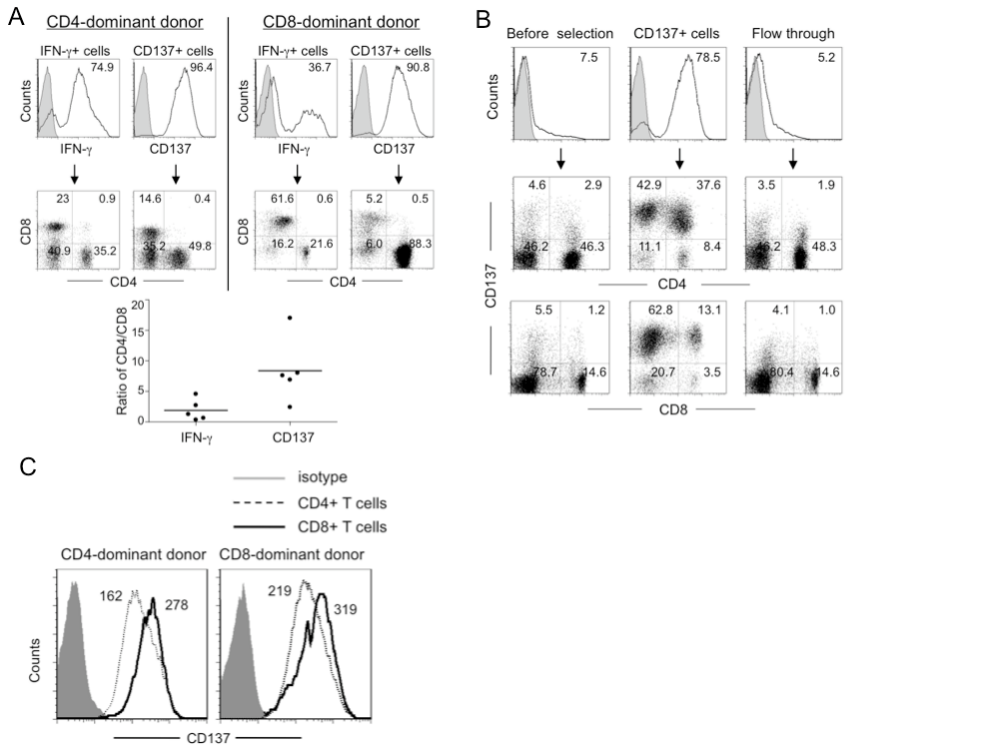
**IFN-γ- or CD137-based enrichment of antigen-specific immune effector cells**. (A) CD4 and CD8 T cell distribution in IFN-γ- or CD137-positive cell population after antigen stimulation. The ratios of CD4 to CD8 T cells of five donors were presented. (B) and (C) CD137 expression in CD4 and CD8 T cells before and after Ab affinity column purification. PBMC were mixed with EBV LMP2 pentadecapeptides and 3–24 hr later, IFN-γ-secreting cells or CD137-positive cells were isolated by using MACS magnetic bead affinity columns as described in Materials and Methods. The cells were stained with PE-conjugated anti-IFN-γ or anti-CD137 Ab and CD3, CD4 and CD8 specific Ab and analyzed with flow cytometry.

The small population of immune effector cells specific for a particular antigen may be isolated based on expression of antigen-specific activation markers. IFN-γ and CD137 have been identified as antigen-specific activation markers. Upon antigen stimulation, the IFN-γ secreting cells can be captured with anti-IFN-γ Ab conjugated with an anti-surface marker Ab. Alternatively, CD137 positive cells can be directly isolated using anti-CD137 Ab. Specific T cell immune response to the peptide library spanning the entire LMP2A sequence has been previously documented. [21] We first established the expression kinetics of IFN-γ and CD137 in PBMC after stimulation with a pool of EBV LMP2A pentadecapeptides, as reports by others, the expression peaked at 6–12 hr for IFN-γ and 24 hr for CD137 (data not shown). [15,22] The IFN-γ- and CD137-expressing cells were isolated using Ab-conjugated magnetic bead affinity columns (Miltenyi Biotech). After examining a large number of donors, we found that the antigen-specific response of an individual could be either CD8 or CD4 T cell dominant, which can be donor- and/or antigen-dependent. Representative results are illustrated in Fig. 1A (a CD4-dominant donor to the left, and a CD8-dominant donor to the right). The results indicated that the CD4/CD8 ratio of the IFN-γ-selected cells correlated with the donor's T cell dominance phenotype. However, the CD137-enriched cells consistently showed an increased CD4 to CD8 ratio regardless of the donor's phenotype (Fig. 1A, bottom); the latter could be due to the increased proportion of CD137 positive CD4 cells in the total population of T cells before and after stimulation (Fig. 1B). However, quantitative analysis of the expression level of CD137 demonstrated that CD8 T cells expressed higher density of surface CD137 than did CD4 T cells in both CD4 and CD8 dominant individuals (see geometric means in Fig. 1C).

### The antigen-activated IFN-γ and CD137 positive immune cells display different surface phenotypes

**Figure 2:**
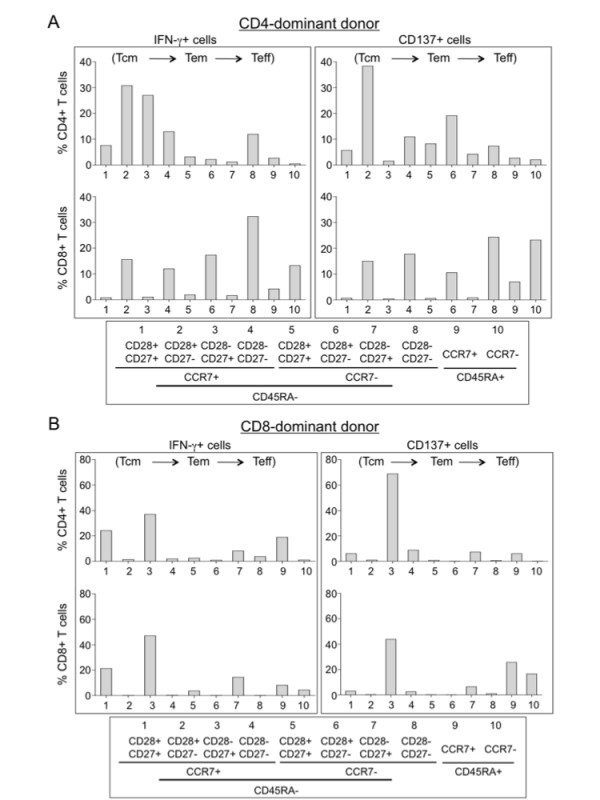
**Phenotype analysis of IFN-γ- or CD137-selected antigen-specific immune cells**. PBMC were stimulated with EBV LMP2A pentadecapeptides and IFN-γ or CD137 positive cells were isolated for analysis. (A) & (B) Memory and effector phenotype analysis based on seven-color flow cytometry with surface staining for CD27, CD28, CD45RA, CCR7, CD4, CD8, CD137 and intracellular staining for IFN-γ immediately after cell isolation without further culture. The percentage of different populations (IFN-γ or CD137 plus CD4 or CD8 gated) of T cells is illustrated for a CD4-dominant donor (A) and a CD8-dominant donor (B); Tcm, central memory T cells (CD27+/-, CD28+, CCR7+, CD45RA-); Tem, effector memory T cells (CD27+/-, CD28+/-, CCR7-, CD45RA-); Teff, terminal effector T cells (CD27-, CD28-, CCR7+/-, CD45RA+).

The phenotype of the immune cells of the two affinity isolation methods has not been characterized in the past due to the limited number of the total harvested cells. We subjected the IFN-γ- and CD137 affinity-purified cells to a multi-color flow cytometry analysis for central memory (Tcm, CCR7+, CD45RA-), effector memory (Tem, CCR7-, CD45RA-), terminal effector (Teff, CCR7-, CD45RA+), differentiation (CD27 and CD28) and migration (CCR7) markers of T cells in addition to CD4, CD8 and IFN-γ (or CD137). We found that there was a trend of increased early-differentiated Tcm and Tem cells in the dominant populations (Fig. 2A and 2B, CD4- and CD8-dominant donors, respectively).

### *Ex vivo *expansion of antigen-activated IFN-γ and CD137 positive immune cells

**Figure 3:**
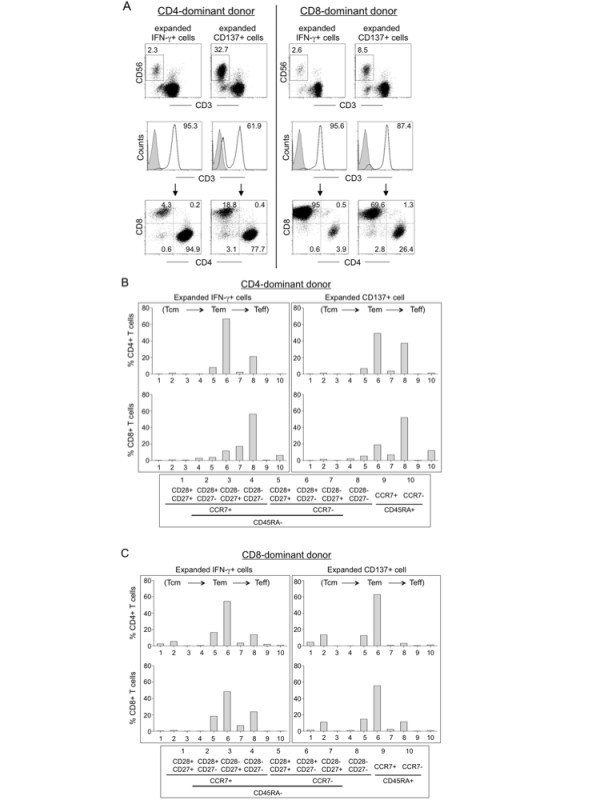
**Phenotype analyses of IFN-γ- or CD137-selected immune effector cells after *ex vivo *expansion**. (A) Phenotype analysis after *ex vivo *expansion. After the rapid selection, the antigen-specific immune cells were cultured for fifteen days with irradiated autologous PBMC as feeder cells. The distribution of CD3, CD4, CD8 and CD56 cell populations was determined and representative FACS graphs are shown. (B) and (C) Memory and effector phenotype analysis based on seven-color flow cytometry with surface staining for CD27, CD28, CD45RA, CCR7, CD4, CD8, CD137 and intracellular staining for IFN-γ. Representative results of a CD4-dominant donor (B) and a CD8-dominant donor (C) are shown.

**Figure 4:**
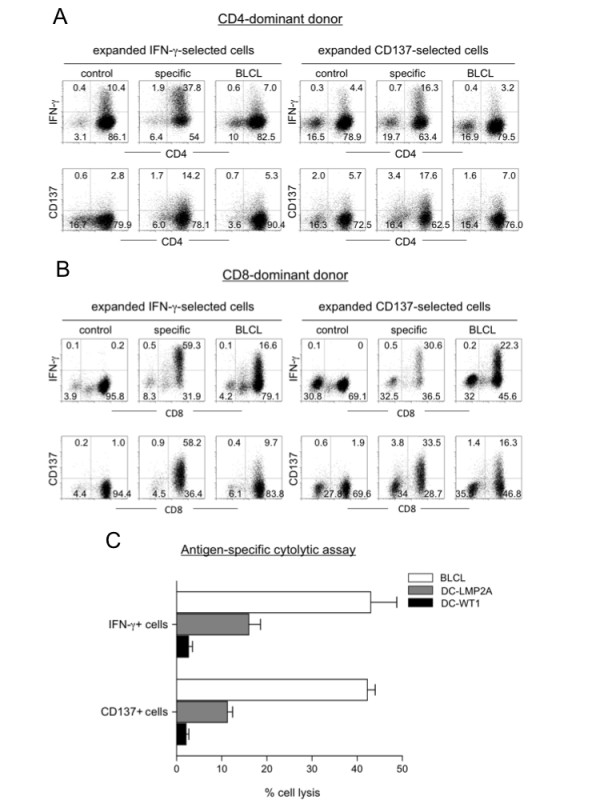
**Effector function analyses of IFN-γ- or CD137-selected immune effector cells after *ex vivo *expansion**. (A) and (B) Flow cytometry analysis of IFN-γ and CD137 expression after restimulation of the culture expanded IFN-γ or CD137 effector cells. The cells were restimulated with different cells as indicated and subjected to antibody staining and flow cytometry analysis; control, WT-1 peptide-pulsed DC; specific, LMP2A peptide-pulsed DC; BLCL, autologous EBV-transformed B cells. (C) Analysis of antigen-specific cytolytic activity. The *ex vivo *expanded IFN-γ- or CD137-enriched cells were incubated with autologous EBV transformed B cells (BLCL), mature DCs loaded with either LMP2A pentadecapeptides (DC-LMP2A) or WT1 pentadecapeptides (DC-WT1, as control) at 1:1 ratio in a cytotoxicity assay based on CFSE labeling as described in Materials and Methods.

To see if the enriched immune effector cells could be further expanded, we cultured them on irradiated autologous feeder PBMC; the cells expanded approximately 600-fold in two weeks (not shown). Before expansion, the CD137 enriched cells contained a higher ratio of CD4 T cells (Fig. 1B, 37.6 vs. 13.1); however, the donor's dominant phenotype was restored after expansion in culture (Fig. 3A). Furthermore, we consistently observed an increased population of CD3^-^CD56^+ ^cells in the CD137+ cell expansion culture compared with the IFN-γ cell expansion culture (Fig. 3A top, 32.7% and 8.5% versus 2.3% and 2.6%, for a CD4-dominant and a CD8-dominant donors, respectively). Compared with the freshly isolated effector cells that contained more memory effector cells (Fig. 2), the cultured cells differentiated toward Tem and (Teff) cells after expansion (Fig. 3B and 3C). Intracellular staining for effector cytokines showed that the expanded IFN-γ and CD137 cells displayed high antigen-specific activities, up to 59% and 34% for IFN-γ and CD137 selected cells, respectively, when restimulated with DC-pulsed with the specific antigens (LMP2A petadecapeptides) or autologous EBV positive BLCL (Fig. 4A and 4B, control: WT-1 peptide-pulsed DC). To demonstrate antigen-specific cytolytic activity, we incubated these cells with different target cells as illustrated in Fig. 4C. The effector cells killed specific target cells with high specificity including autologous BLCL and LMP2A peptide-pulsed DC, but not control WT-1 peptide-pulsed DC (Fig. 4C, effector to target ratio 1:1).

### *Ex vivo *expansion of antigen-specific immune effector cells with dendritic cells (DC)

**Figure 5:**
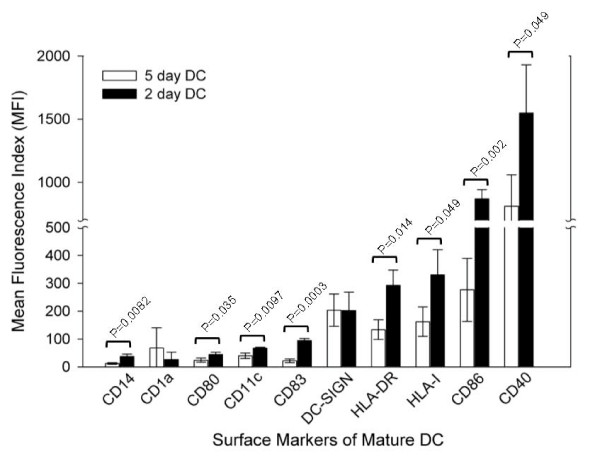
**Phenotype comparison of 5 day versus 2 day mature DC**. Surface markers related to antigen presentation function were stained with multi-color fluorochrome-labeled Ab and analyzed with FACSAria. Data represent MFI of mature DC generated from three healthy donors with p value calculated.

Antigen-specific immune effector cells can also be generated through DC activation. The latter protocol includes a DC preparation step, followed by antigen exposure and lymphocyte coculture. For the preparation of DC, we compared the 2 day and the 5 day protocols. [23] We analyzed the surface markers for plastic-adherent monocytes and mature DC with flow cytometry (data shown in Additional file 1), and analyzed phenotypes of the 2 day and the 5 day mature DC (Fig. 5). The 2 day DC expressed higher levels of class I/II MHC (HLA-I and HLA-DR), costimulatory molecules (CD86 and CD40) and maturation marker CD83. We also found that the 2 day DC induced primary and secondary immune response against viral or cancer antigens at efficiencies equal to or better than the 5 day DC (manuscript in preparation). Therefore, the 2 day DC protocol was adopted for later experiments.

**Figure 6:**
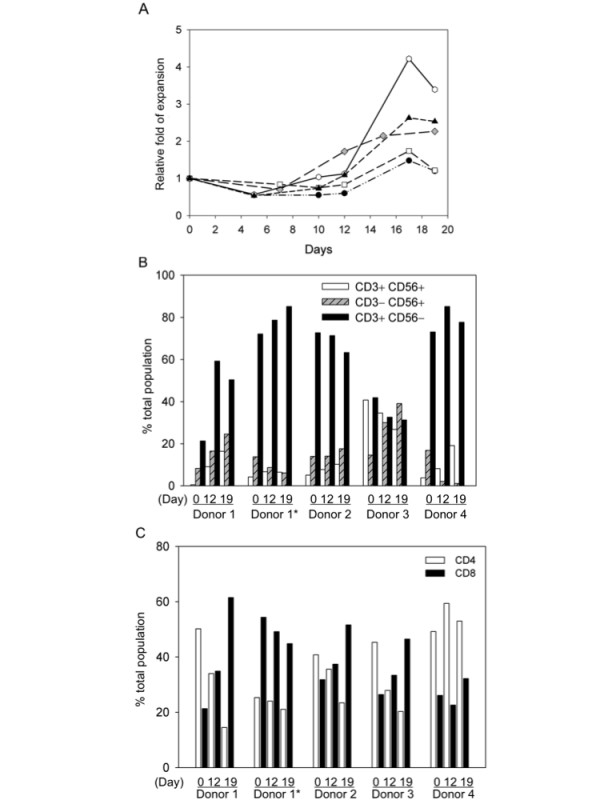
***Ex vivo *expansion and phenotype analyses of DC-activated immune effector cells**. EBV LMP2A-specific effector T cells were generated by stimulation of non-adherent PBMC with DC pulsed with LMP2A pentadecapeptides. (A) Growth kinetics of DC-activated immune effector cells. The viable cells were counted with trypan blue staining at different time points after coculture and the growth curves of 5 samples are shown. (B) and (C) CD3, CD56, CD4 and CD8 phenotype analysis. The DC-activated cells from day 0, 12 and 19 were stained with antibodies against CD4, CD8, CD3 and CD56 and analyzed with flow cytometry. The percentages of different lymphocyte subsets were analyzed and shown in bar graphs. Donor 1*, PBMC collected at a different time point from donor 1.

**Figure 7:**
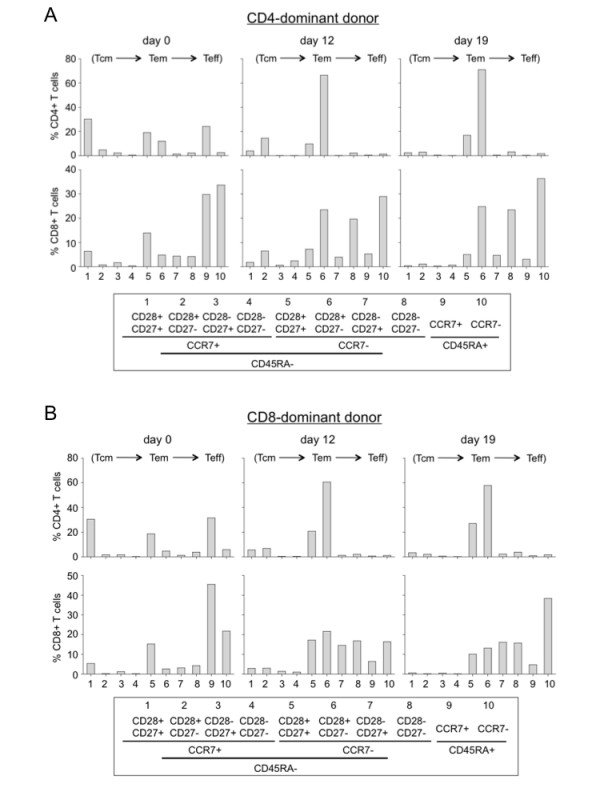
**Memory and effector T cell analyses of DC-activated immune effector cells**. (A) & (B) Memory and effector phenotype analysis of a CD4 dominant donor (A) and a CD8 dominant donor (B). The DC-activated cells from day 0, 12, and 17 after coculture were analyzed for CD3, CD4, CD8, CD27, CD28, CD45RA, and CCR7 using a seven-color panel of fluorochrome-labeled Ab with FACSAria. The cells were gated for CD3 and CD4 or CD8 as total cells for the percentage analysis. One representative flow graph of 5 performed experiments is presented.

The 2 day DC were pulsed with the pooled LMP2A pentadecapeptides, irradiated and cocultured with autologous lymphocytes at a ratio of 1:20. The cell number usually decreases around day 5 after coculture, followed by an increase of a few fold around day 17, suggesting a loss of non-specific cells followed by expansion of antigen-specific cells (see representative growth curves in Fig. 6A). Flow cytometry analysis of the DC-activated cells in culture from four different donors at day 0, 12 and 19 indicated that most donors generated CD3+CD56- T cells, but some generated a large proportion of CD3-CD56+ NK cells and CD3+CD56+ cells (e.g. donor 3, Fig. 6B). The relative ratios of CD4+ T cells, CD8+ T cells, NK cells and CD3+CD56+ cells in the coculture appeared to be donor-dependent (Fig. 6B and 6C). Furthermore, phenotype analysis with multi-color flow cytometry demonstrated that the *ex vivo *expanded cells contained mostly Tem and Teff cells (Fig. 7A and 7B, representative of CD4- and CD8-dominant donors).

### The DC-activated immune cells display antigen-specific effector functions

**Figure 8:**
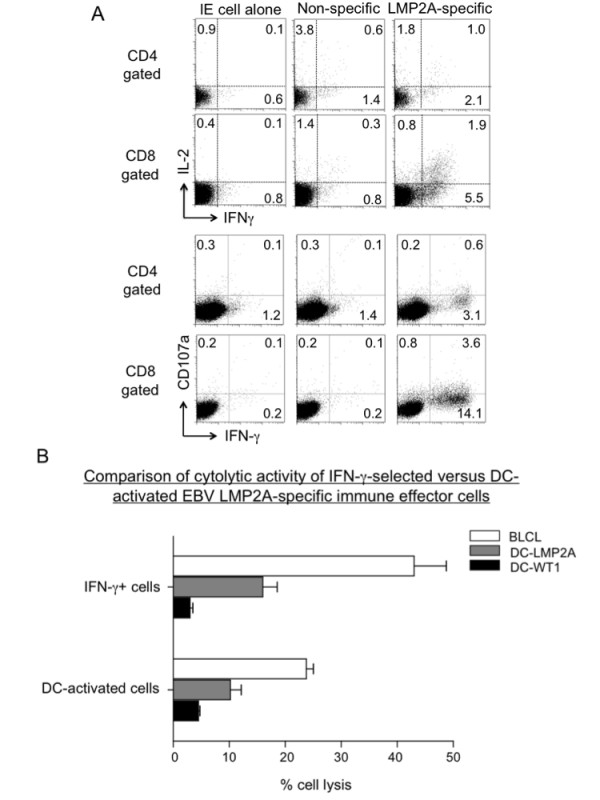
**Functional analyses of DC-activated and *ex vivo *expanded immune effector cells**. EBV LMP2A-specific effector cells were generated by stimulation of non-adherent PBMC with DC pulsed with LMP2A pentadecapeptides. (A) Analysis of antigen-specific effector cytokines and CD107a expression. The DC-activated immune cells from day 19 coculture were stimulated with autologous DC pulsed with LMP2A peptides, WT1 peptides (non-specific control) or no stimulation (IE cell alone) for 6 hours. The cells were stained with Ab against CD4, CD8, CD107a and IFN-γ. Flow cytometry analyses of IL-2, IFN-γ and CD107a-positive cells in CD4-gated or CD8-gated populations were illustrated as representative of five experiments. (B) Comparison of cytolytic function of IFN-γ-selected (same donor as in Fig. 4C) versus DC-activated LMP2-specific immune effector cells. The LMP2A-specific effector cells were mixed with target cells including autologous BLCL, DC-LMP2A, or control DC-WT1 at 1:1 ratio and analyzed for cytolytic activity based on the CFSE-labeling method as described in Materials and Methods.

We next evaluated the DC-activated immune cells for antigen-specific effector functions based on intracellular staining of IFN-γ, IL-2, and the degranulation marker CD107a after restimulation with peptide-loaded DC (Fig. 8A). For direct comparison, the same donor whose cells were analyzed for the IFN-γ-selected effector functions (Fig. 4B) was chosen. Analyses of the immune effector cells without further stimulation (IE cell alone), or stimulated with non-specific peptides (Non-specific) or with the LMP2A pentadecapeptides (LMP2A-specific) demonstrated LMP2A-specific expression of IFN-γ, IL-2 and CD107a in the CD8 T cells, and to a lesser extent, the CD4 T cells (Fig. 8A top: IL-2 and IFN-γ, and bottom: CD107a and IFN-γ). The antigen-specific effector function was further confirmed with the CFSE-based proliferation assay, as well as MHC-peptide pentamer specific for the LMP2A epitope-specific T cell receptors of the CD8 T cells (data not shown).

To demonstrate antigen-specific killing, we directly compared the DC-activated cells to the IFN-γ captured cells (as shown in Fig. 4C) using an *in vitro *cytolytic assay (Fig. 8B). At an effector to target cell ratio of 1:1, the immune cells effectively killed autologous BLCL and LMP2A-pulsed DC (DC-LMP2A), but not DC pulsed with control WT1 pentadecapeptides (DC-WT1). Therefore, effector function analyses including IFN-γ release and cytotoxicity assays suggest that the DC-activated cells had lower activities than the expanded IFN-γ effector cells.

## Discussion

Adoptive immune cell therapy has shown great promise in treating viral diseases and melanoma. [2,6] Continued efforts are focused on the generation of sufficient amount of antigen-specific immune cells and optimal conditioning of immune homeostasis in patients in order to achieve a sustained *in vivo *immune surveillance. [24-26] Here, we compared three *ex vivo *immune cell preparation protocols and phenotypically and functionally characterized these cells. The rapid protocols based on IFN-γ and CD137 selection generate a small number of antigen-specific effector cells with high percentage of central memory T cells in a very short period of time. The DC-activation protocol generates more immune cells, albeit, with more differentiated phenotype and reduced proportion of antigen-specific effector cells.

Based on analyses of a large number of donors, we found that individual response to a given antigen could be either CD4- or CD8-dominant, which is antigen- and donor-dependent. Immune effector cells isolated based on IFN-γ expression displayed a CD4 or CD8 bias consistent with the donor's immune dominance. However, antigen-specific CD137 positive cells showed a higher ratio of CD4 effector cells regardless of the subject's immune phenotype; this is in contrast to previous reports that emphasize the induction of CD8 effector cells after CD137 enrichment. [15,22,27] We did, however, show that CD8 T cells displayed higher density of CD137 than did CD4 T cells. It is well documented that CD137 costimulation promotes both CD4 and CD8 T cell expansion and long term memory. [28-30] Our finding that more CD4 T cells than CD8 T cells are detected in the CD137-positive cell population suggests a rapid induction of CD137 in the memory T helper repertoire immediately after antigen stimulation. Although the enriched CD137 immune cells contained a higher CD4 T cell ratio, further expansion in culture restored the donor's original dominant phenotype, with a higher CD3-CD56+ NK cell population than those found in the expanded IFN-γ enriched immune cells (Fig. 3A). This result suggests that IFN-γ is a more restricted adoptive immune response marker and represent less of an innate immune marker as does CD137.

The *ex vivo *DC-activation protocol generated different ratios of CD4, CD8 and NK cells in culture, which again, appeared to be donor-dependent. Whether the immune dominance has any effect on *in vivo *efficacy of the cultured immune effector cells awaits further investigation. As CD4 T cells are important for the maintenance of long-term anti-viral CD8 T cell memory [31], therapeutic immune cells should include polyclonal CD4 and CD8 T cells. Both IFN-γ and CD137 selection approaches generated increased number of memory type of cells representative of polyclonal CD4 and CD8 T cells that may have increased proliferation potential after infusion. Although the antigen-specific memory T cells from PBMC may be low; for examples, the average yield of LMP2A-specific IFN-γ positive immune effector cells from healthy EBV-seropositive donors is only 0.22 ± 0.13% (n = 6, after two rounds of affinity column purification), they can be expanded to more than two orders of magnitude in culture in two weeks and maintain their high antigen specificity.

It is evident that the LMP2A pentadecapeptides efficiently activate both CD4 and CD8 T cells in a short exposure period (3–13 hr). This was surprising since CD8 T cells are activated through class I MHC loaded with short 9–11 amino acid peptide epitopes, different from CD4 T cells, which are activated through class II MHC loaded with 12–15 amino acid peptide epitopes. The pentadecapeptide antigens apparently activated CD8 T cells with high efficiency through cross-presentation. This has been confirmed with various pentadecapeptide antigens (unpublished). The processing of class II MHC peptides into class I epitopes for cross-presentation to CD8 T cells appears to be highly efficient with both the IFN-γ and the CD137 protocols, as with the DC-activation method. The detailed molecular mechanism of the efficient cross-presentation requires further investigation.

To assess differentiation and maturation status of the *ex vivo *generated T cells, we applied multi-color flow cytometry to detect differentiation and homeostatic marker CD45RA, trafficking marker CCR7, and costimulatory marker CD27 and CD28. [32,33] It is not surprising that both the IFN-γ- and the CD137-enriched antigen-specific effector cells displayed more memory markers than did the DC coculture-expanded cells. While preserving Tcm cells is critical to *in vivo *therapeutic efficacy,[7,34,35] clinical studies have proven that *ex vivo *expanded effector cells can persist many years after infusion. [36] Clinical benefits of these different protocols will require detailed evaluation in a large cohort of patients.

The differentiation status of the *ex vivo *generated immune cells may contribute to their *in vivo *therapeutic efficacy. Homeostasis of antigen-specific memory cells can vary depending on antigen source, the immune milieu and individual donor. It is known that Tcm cells are mainly located in lymphoid tissues and Tem cells are distributed in diverse non-lymphoid sites including lung, liver and intestine. [37] In addition, bone marrow has been shown to embrace increased number of anti-cancer or anti-virus memory T cells. [38-40] After *ex vivo *expansion, however, wherever the T cells come from, they tend to bestow exhausted proliferation and replicative senescence associated with down-regulation of anti-apoptotic protein Bcl-2 and Bcl-xL, and decreased telomere length. [33,34,41] Modification of antigen presentation protocol and culture condition may help overcome the immune cell exhaustion problem. [9,42]

For patients with acute infections or illness, direct isolation of antigen-specific immune cells from partly HLA-matched healthy donors represents an attractive emergency approach to obtain therapeutic cells. [43,44] This approach offers several advantages including a shortened handling time and increased proliferation potential *in vivo*. Although the number of immune cells is limited with the direct isolation approach, clinical evidence supports that only a small number of such immune cells, in the range of 10^3^-10^4^/kg body weight, is sufficient to attain therapeutic efficacy in transplant patients. [13,45,46]*Ex vivo *expansion of immune cells, nevertheless, may be necessary for patients with a compromised immunity. [47,48]

## Conclusion

The two rapid immune cell isolation methods generate functional effector cells in less than 24–48 hr suitable for emergency immune cell preparation. On the other hand, the DC-activation method expands antigen-specific immune effector cells while effectively reduce the number of non-specific cells. Depending on clinical needs, for examples, the urgency for treatment, patient's body weight (e.g. less cells are needed for pediatric patients), or patient's immune cell proliferative potential *in vivo*, the method of immune cell preparation may differ. Our data indicate that IFN-γ selection followed by *ex vivo *expansion represents the best approach for the generation of high amount of antigen-specific immune effector cells. Further efforts to overcome immune tolerance and expand antigen-specific immune cells with prolonged *in vivo *persistence are critical to the success of immune cell therapy.

## List of abbreviations

IFN-γ: interferon-gamma; IL: interleukine; DC: dendritic cell; CTL: cytotoxic T lymphocyte; MHC: major histocompatibility complex; Ab: antibody; Ag: antigen; TCR: T cell receptor; TNF: tumor necrosis factor; BLCL: B lymphoblastoid cell line; EBV: Epstein-Barr virus; LMP2A: late membrane protein 2A; CMV: cytomegalovirus; ICCS: intracellular cytokine staining; CFSE: carboxy-fluorescein diacetate succinimidyl ester.

## Competing interests

YH, YL, YH and YW are employees of Vectorite Biomedica Inc. LJC is consultant to a biotech company.

## Authors' contributions

All authors are accountable for the integrity of the research results; Chang is responsible for the conception of the research and Han, Huang, Liang, Ho and Wang are responsible for the execution and for data collection; Chang is responsible for initial drafting and revisions of the manuscript.

## Supplementary Material

Additional file 1 **Phenotype analysis of monocytes and 2 day and 5 day mature DC**. Surface markers related to antigen presentation function were analyzed using fluorochrome-labeled Ab. The light-colored lines in the FACS graphs represent control Ab and the numbers represent geometric means with percentages shown in parentheses. Representatives of two monocyte experiments and three DC experiments are illustrated. Click here for file
