# Supplementary figures and images for: Phenotype and functional evaluation of ex vivo generated antigen-specific immune effector cells with potential for therapeutic applications
Source: J Hematol Oncol. 2009 Aug 6;2:34. doi: 10.1186/1756-8722-2-34 (PMC2729746; doi:10.1186/1756-8722-2-34)

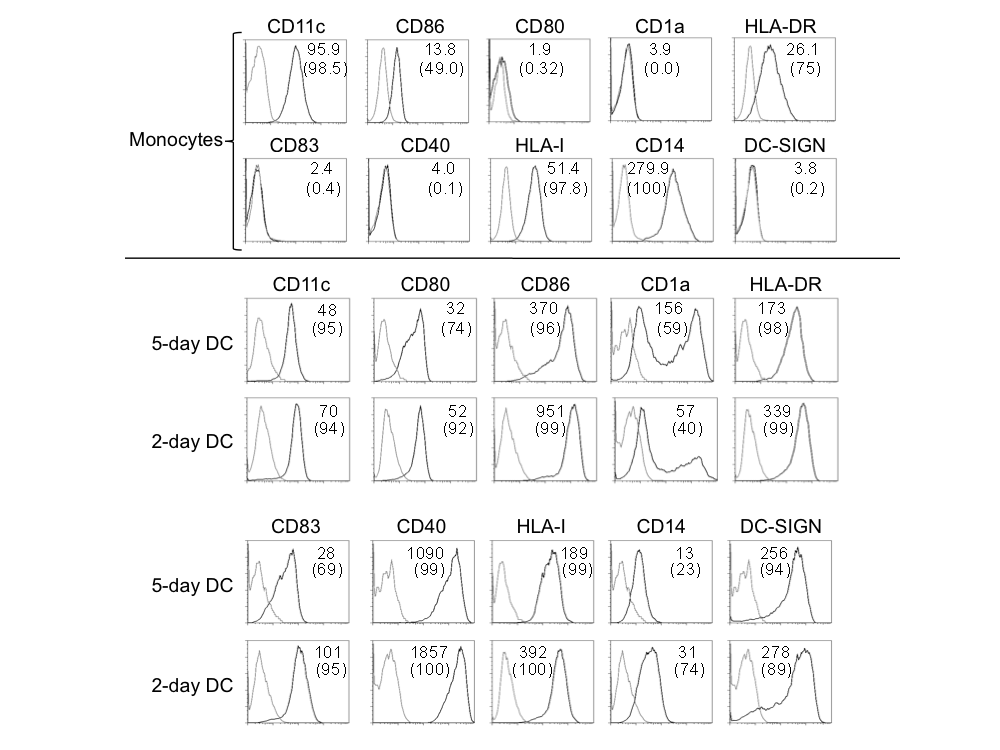

Supplement: Additional file 1 — Phenotype analysis of monocytes and 2 day and 5 day mature DC. Surface markers related to antigen presentation function were analyzed using fluorochrome-labeled Ab. The light-colored lines in the FACS graphs represent control Ab and the numbers represent geometric means with percentages shown in parentheses. Representatives of two monocyte experiments and three DC experiments are illustrated. [file 1756-8722-2-34-S1.tiff]
